# Association between hemoglobin glycation index and 28-day all-cause mortality in acute myocardial infarction patients: Analysis of the MIMIC-IV database

**DOI:** 10.1371/journal.pone.0330819

**Published:** 2025-09-02

**Authors:** Yue Lv, Lingchen Wei, Ziyue Wang, Zichuan Mu, Jianlin Wu

**Affiliations:** College of Traditional Chinese Medicine, Shandong University of Traditional Chinese Medicine, Jinan, Shandong, China; Tongji University Tenth People's Hospital: Shanghai Tenth People's Hospital, CHINA

## Abstract

Acute myocardial infarction (AMI) substantially fuels the worldwide escalation in both morbidity and mortality. The hemoglobin glycation index (HGI) is linked to a range of undesirable outcomes, but its relationship with short-term outcomes in AMI patients has not been explored. This study analyzed data from 1008 first-time ICU AMI patients in the MIMIC-IV 3.1 database. To calculate the HGI, a linear regression equation was developed based on fasting glucose (FPG) and glycosylated hemoglobin (HbA1c), and patients were classified into four quartile groups. The main outcome of interest was 28-day ICU mortality, with the secondary outcome being 28-day in-hospital mortality. Kaplan-Meier survival analysis revealed that the Q1 group (low HGI) exhibited significantly higher mortality rates compared to the other groups. In a well-adjusted Cox proportional hazards model, low HGI was drastically linked with 28-day ICU mortality and 28-day in-hospital mortality. Restricted cubic spline (RCS) analysis revealed a U-shaped association between HGI and outcome events, mainly characterized by a correlation between low HGI and poor outcomes. Subgroup studies revealed that the association between HGI and endpoints was constant across subgroups. Machine learning models, including Boruta and SHAP, confirmed HGI’s predictive value for short-term adverse outcomes. This shows that HGI could be a useful indicator of short-term mortality in AMI patients.

## Introduction

Globally, acute myocardial infarction (AMI) has become one of the key factors causing disease and death. It is highly prevalent and lethal, and every year a large number of people suffer health risks and even lose their lives as a result of the disease [1]. Nearly 50% of AMI patients in the United States have been admitted to the ICU in the last 20 years. From 2013 to 2016, around 7.9 million U.S. adults suffered from AMI, and 114,023 individuals died from it in 2015 [2–4]. More aggressive therapy for the prognosis of patients with AMI is needed; however, there are just a few relevant studies.

Diabetes mellitus is an important underlying condition in AMI. As a result of chronic hyperglycemia, vascular endothelial cells in diabetic patients are damaged, accelerating the process of atherosclerosis, which in turn increases the risk of acute myocardial infarction. Several studies have demonstrated that diabetes not only increases the likelihood of AMI but also leads to higher mortality and poorer prognosis [5,6]. Several studies have indicated that hyperglycemia is commonly observed after AMI, and maintaining stable blood glucose levels has significant benefits for the long-term health of AMI patients [7–9]. Although HbA1c is widely recognized as a reliable indicator of glycemic control and is considered the gold standard for assessing long-term glycemic control in diabetic patients [10]. However, evidence suggests that there is a significant deviation between measured HbA1c and predicted HbA1c levels derived from fasting blood glucose (FPG) in certain populations [11]. The HGI can quantify this discrepancy and may reflect acute metabolic disorders that traditional indicators like HbA1c fail to capture [12]. HGI indicates the difference between actual HbA1c measurements and those predicted by a linear regression equation derived from average blood glucose levels [13]. Numerous studies have discovered that HGI is substantially related to the prevalence of cardiovascular disease (CVD) and microvascular issues in patients with diabetes [14]. However, research on the correlation between HGI and the short-term deterioration of AMI patient prognosis is relatively limited. Therefore, this study utilizes data from the fourth phase of the Medical Information Mart for Intensive Care (MIMIC-IV) to construct a linear regression equation for calculating HGI and to investigate its relationship with unfavorable outcomes among patients with AMI. By this study, we aim to deliver new detection and intervention indicators for patients with AMI, eventually decreasing the risk of death in these patients. Moreover, understanding the relationship between HGI and AMI prognosis may provide insights into the management strategies for diabetic patients with AMI, as the blood sugar control status of diabetic patients is closely related to HGI.

## Methods

### Data source

This study used the MIMIC-IV 3.1 database to access data on ICU patients admitted to Beth Israel Deaconess Medical Center (BIDMC) from 2008 through 2022 [15]. The authors (LY) were granted database access (certificate number: 66558897).

### Inclusion and exclusion criteria

Inclusion criteria:

(1) individuals aged from 18 to 90 years;
(2) diagnosed with AMI;
(3) initial admission to the ICU.

Exclusion criteria:

(1) ICU status of fewer than 24 hours;
(2) absence of serum glucose and glycated hemoglobin data from initial laboratory tests;
(3) only data from the first admission were considered for patients with multiple admissions to the ICU;
(4) Age < 18 years and age > 90 years;
(5) absence of the SOFA score, APS III score and SAPS II score.

**Fig 1:**
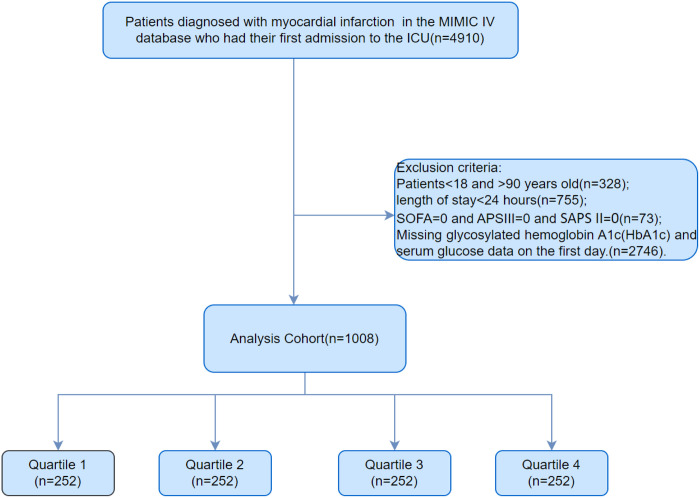
Inclusion and exclusion criteria flow chart of the study population. 1008 patients were enrolled.

Finally, 1008 patients with AMI were enrolled in MIMIC-IV (Fig 1). The study used the International Classification of Diseases (ICD-10 and ICD-9 codes) to describe AMI and comorbidities.

### Data extraction

Structured Query Language (SQL) was used alongside PostgreSQL 16.5 and Navicat Premium 16 for raw data extraction. Variables gathered in this research comprised: (1) demographic data: age, gender, BMI; (2) comorbid conditions: hypertension, Diabetes I, Diabetes II, HLD, CHD, CKD, AKI, HF; (3) laboratory indicators: WBC, RBC, HGB, PLT, HbA1c, albumin, FPG, sodium, potassium, CK; (4) severity of illness scores: SOFA, APSIII, SAPS II.

### Statistical analysis and model development

A linear regression model between FPG and HbA1c was constructed using data from all included patients. On the basis of this, predicted HbA1c = 0.0075 × FPG (mg/dL) + 5.18. HGI = measured HbA1c-predicted HbA1c [16]. HGI was categorized into four quartiles: Q1 (HGI < −0.81), Q2 (−0.81 ≤ HGI < −0.35), Q3 (−0.35 ≤ HGI < 0.32), and Q4 (HGI ≥ 0.32). 28-day ICU mortality was the primary outcome, and 28-day in-hospital mortality was the secondary outcome.

The multiple imputation was employed to address the variables with a missing data rate below 20%, whereas those with rates greater than 20% were removed. The variables that fit the normal distribution were assessed using an analysis of variance. The Mann-Whitney U test was used to compare the variables that did not fit the normal distribution. Percentages were used to express categorical variables. Additionally, Kaplan-Meier survival curves were employed for evaluation in order to examine the variations in survival rates across the four groups over a 28-day period. The COX regression model was constructed using the Q3 group as a baseline and adjusted for variables so as to assess whether HGI was an independent risk factor for outcome events and to generate hazard ratio (HR) and 95% confidence intervals (95% CI). Model I was corrected for BMI; Model II was calibrated to BMI and comorbidities such as hypertension; and Model III was modified for BMI, comorbidities, SOFA score, SAPS II score, APS III score, and laboratory indicators such as leukocytes. The connection between the continuous variable HGI and outcome events was determined using RCS. Subgroup analyses were utilized to increase the accuracy and reliability of the results. The study was statistically evaluated using R Studio 4.3.3, with a bilateral P value < 0.05 indicating statistical significance.

This study employed six algorithms to construct predictive models: Categorical Boosting (CatBoost), Decision Tree Learner Plus (DecisionTree), K-Nearest Neighbor Learner Plus (KNN), Logistic Classification Learner Plus (Logistic), Random Forest Learner Plus (Random Forest), and eXtreme Gradient Boosting (XGBoost). Hyperparameter tuning was conducted during the model development process.The data were separated into a training set and a validation set for model building and evaluation, respectively. The best-performing model is identified as the final model. Model performance was assessed using the ROC curve and its corresponding area under the curve (AUC), with the best-performing model being identified as the final model. In this study, Boruta’s algorithm, constructed based on the CatBoost algorithm, was used for feature filtering to ascertain the significance of each feature in the prognosis model. This algorithm utilizes the concepts of “shadow features” and “binomial distribution.” This approach employs the ideas of “shadow features” and “binomial distribution,” as well as repeated bootstrap resamples of the dataset to create various random forests. Within each random forest, feature importance is calculated using variable importance metrics. Boruta’s algorithm is intended to offer a stochastic measure of feature relevance by using “shadow features” as a comparison to the originals. If a feature’s importance significantly exceeds that of its corresponding “shadow feature,” it is marked as “important” (red region); otherwise, it is marked as “unimportant” (yellow region) [17]. The SHAP approach increases the model’s interpretability. The summary plot is an interpretation of the predictions for the entire sample: one method is to take the average absolute value of the SHAP values for each feature and generate a normalized bar graph describing the importance of each covariate in developing the final predictive model. The other is to simply plot the SHAP values of each feature for each sample using a scatterplot, where the colors indicate the link between the magnitude of the feature values and the relative values [18].

## Results

### Baseline characteristics

The study involved 1008 patients with AMI. S1 Table shows baseline data sorted by HGI quartile (for a detailed flow diagram see Supplementary Table). The patients had an average age of 67.20 years, with 687 males (68.15%) and 321 females (31.85%). The prevalence of hypertension was 36.11% (n = 364), acute kidney injury (AKI) was 46.23% (n = 466), type 2 diabetes was 38.89% (n = 392), type 1 diabetes was 2.68% (n = 27), heart failure was 2.68% (n = 27), chronic kidney disease (CKD) was 24.01% (n = 242), and hyperlipidemia was 52.28% (n = 527). All patients were diagnosed with myocardial infarction (MI) and coronary heart disease (CHD) (100.00%). Patients were separated into four groups depending on the HGI levels: Q1 (n = 252, HGI < −0.81), Q2 (n = 252, −0.81 ≤ HGI < −0.35), Q3 (n = 252, −0.35 ≤ HGI < 0.32), and Q4 (n = 252, HGI ≥ 0.32). Compared to the lower HGI groups, the higher HGI groups had higher BMI, higher counts of RBC and HbA1c, and a greater proportion of patients with AKI, CKD, Diabetes I, Diabetes II, and HLD. In contrast, the lower HGI groups had a higher proportion of patients with hypertension and HF. Additionally, higher HGI groups and lower HGI groups exhibited higher disease severity scores (APS III and SAPS II), WBC counts, and FPG compared to the median HGI group.

### Clinical outcomes

**Fig 2:**
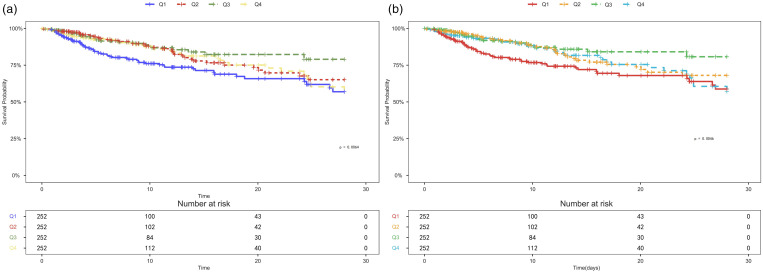
Kaplan-Meier all-cause mortality survival analysis curve. (A) Kaplan–Meier curves depict the probability of ICU mortality by group at 28 days and (B) hospital mortality by group at 28 days.

Fig 2 depicts the findings of the Kaplan-Meier analysis of outcome events, which compared the survival differences between the four different HGI groups. A detailed analysis revealed that the Q1 group had considerably higher mortality rates than the other groups (P < 0.05, statistically significant), while the Q3 group maintained the lowest mortality throughout the observation period. Although there was some convergence among the Q1, Q2, and Q4 groups in later phases, the cumulative mortality in Q1 remained persistently higher than that in Q3. Furthermore, mortality rates in both the Q1 and Q4 groups gradually increased over time. These data suggest that HGI could serve as an independent predictor of short-term outcomes in AMI patients. Low HGI values were associated with increased mortality risk, while moderate HGI levels were associated with better survival outcomes. The findings highlight the potential clinical utility of HGI as a risk stratification tool in practice.

By comparing the baseline information of patients, it was determined that Group Q3 (−0.35 ≤ HGI < 0.32) had the lowest mortality rate. Based on this, we established a Cox proportional hazards regression model using Group Q3 as the reference group (Table 1). The results showed that in the unadjusted Cox proportional hazards model, Group Q1 (HGI < −0.81) showed a major link with 28-day ICU mortality (HR, 2.13 [95% CI 1.33, 3.42], P = 0.002) and 28-day in-hospital mortality (HR, 2.25 [95% CI 1.38, 3.68], P = 0.001). Although Group Q4 had a greater mortality risk, the relationship was not statistically significant. In Model 1 (adjusted for BMI), Group Q1 was still significantly linked with 28-day ICU mortality (HR, 2.05 [95% CI 1.27, 3.30], P = 0.003) and 28-day in-hospital mortality (HR, 2.17 [95% CI 1.32, 3.55], P = 0.002). Notably, the correlation with 28-day mortality risk was somewhat lower than in the unadjusted model. In Model 2 (adjusted for BMI, hypertension, AKI, etc.) and Model 3 (additional adjustments for WBC, HGB, RBC, etc. in Model 2), Group Q1 remained significantly associated with outcome events. All trend tests (quartiles) showed significant differences.

**Table 1:** A multivariate Cox regression analysis of HGI with survival in myocardial infarction patients.

HGI	Unadjusted model		Adjusted model 1		Adjusted model 2		Adjusted model 3	
	HR(95% CI)	P value	HR(95% CI)	P value	HR(95% CI)	P value	HR(95% CI)	P value
ICU mortality								
HGI(<−0.81)	2.13(1.33,3.42)	**0.002**	2.05(1.27,3.30)	**0.003**	2.02(1.25,3.28)	**0.004**	1.70(1.01,2.85)	**0.045**
HGI(−0.81, −0.35)	1.29(0.77,2.15)	0.342	1.23(0.73,2.07)	0.431	1.27(0.75,2.15)	0.369	1.09(0.65,2.05)	0.742
HGI(−0.35,0.32)	Ref		Ref		Ref		Ref	
HGI(>0.32)	1.44(0.87,2.39)	0.152	1.43(0.87,2.37)	0.158	0.93(0.54,1.59)	0.793	0.82(0.43,1.58)	0.559
HGI group trend	0.05		0.049		0.043		0.041	
in-hospital mortality								
HGI(<−0.81)	2.25(1.38,3.68)	**0.001**	2.17(1.32,3.55)	**0.002**	2.13(1.29,3.51)	**0.003**	1.79(1.05,3.05)	**0.033**
HGI(−0.81, −0.35)	1.33(0.76,2.27)	0.303	1.27(0.74,2.18)	0.378	1.31(0.76,2.25)	0.337	1.11(0.63,1.93)	0.724
HGI(−0.35,0.32)	Ref		Ref		Ref		Ref	
HGI(>0.32)	1.54(0.92,2.58)	0.103	1.53(0.91,2.57)	0.108	1.05(0.60,1.83)	0.863	0.91(0.47,1.79)	0.79
HGI group trend	0.032		0.031		0.027		0.024	

Unadjusted model: no variables were adjusted.

Model 1:Adjusted for BMI.

Model 2:Adjusted for BMI, hypertension, AKI, CKD, diabetes II, diabetes I, Hyperlipidemia, heart failure, and MI.

Model 3: Adjusted for BMI, hypertension, AKI, CKD, diabetes II, diabetes I, Hyperlipidemia, heart failure, MI, WBC, HGB, RBC, HbA1c, albumin, sodium, and CK.

### Restricted cubic spline

**Fig 3:**
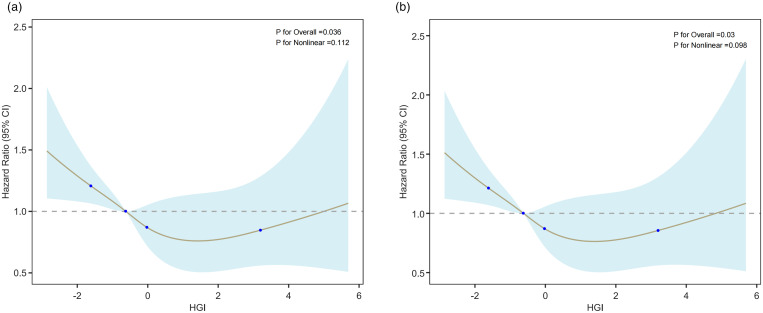
Restricted cubic spline(RCS) curve for the HGI hazard ratio. (A) RCS curve for ICU mortality at 28 days, and (B) RCS curve for hospital mortality at 28 days. Curves show adjusted hazard ratios, with shaded ribbons indicating 95% CI and a horizontal dashed line at a HR of 1.

Fig 3 illustrates the correlation between HGI and mortality. The RCS analysis for 28-day ICU mortality (Fig 3A) and 28-day in-hospital mortality (Fig 3B) both indicate a U-shaped relationship between HGI and endpoints (P for overall < 0.05, statistically significant). Furthermore, P for nonlinearity > 0.05 indicates that the relationship is not statistically different from linearity. When the HGI level is low, the mortality rate increases rapidly with the increase of HGI, and the mortality rate remains relatively stable within a certain range before slowly increasing with the increase of HGI, indicating that both low HGI and high HGI are associated with an increase in mortality risk, with patients in the medium HGI range having the lowest mortality risk.

### Subgroup forest plot

**Fig 4:**
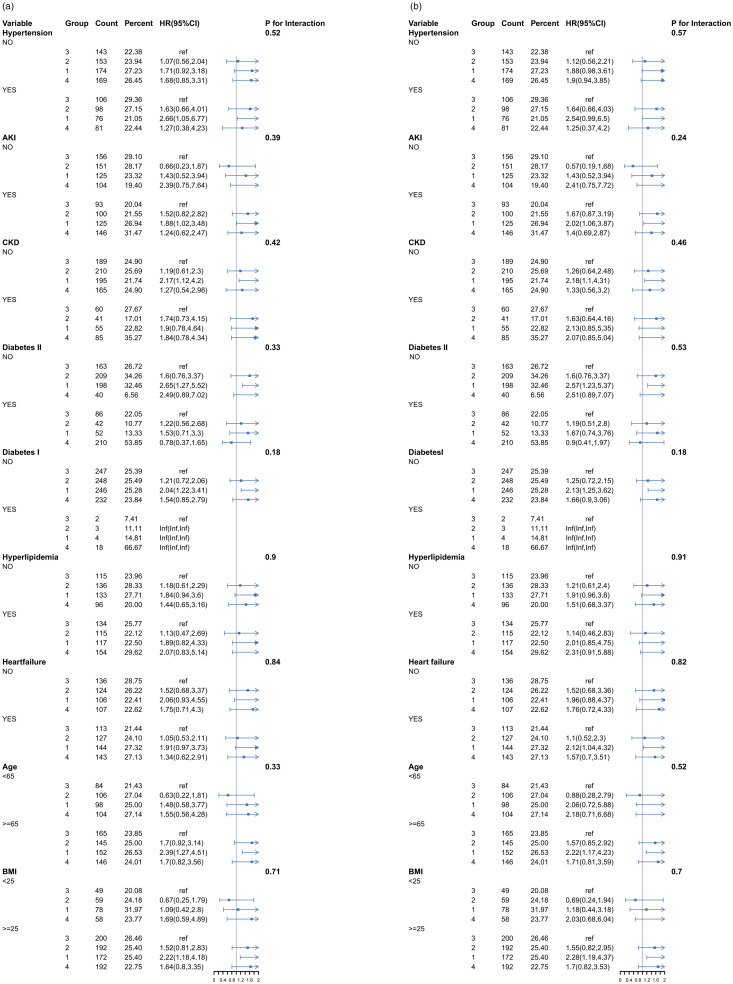
Subgroup forest plot. (A) subgroup forest plot curve for ICU mortality at 28 days, and (B) subgroup forest plot curve for hospital mortality at 28 days.

Additionally, we conducted subgroup analyses based on age, BMI, hypertension, AKI, CKD, diabetes I, diabetes II, HLD, and heart failure to assess patient prognosis. In the subgroup analysis with 28-day ICU mortality (Fig 4A), the risk of death in patients aged 65 years and older, hypertensive patients, non-diabetic patients, AKI patients, non-CKD patients, and patients with a BMI ≥ 25 were closely related to the Q1 group (HGI < −0.81). In the subgroup analysis with 28-day in-hospital (Fig 4B), the risk of death in patients aged 65 years and older, patients with AKI, patients without CKD, patients without diabetes, patients with heart failure, and patients with a BMI of ≥ 25 were strongly associated with the Q1 group (HGI < −0.81).

### Receiver operator characteristic

**Fig 5:**
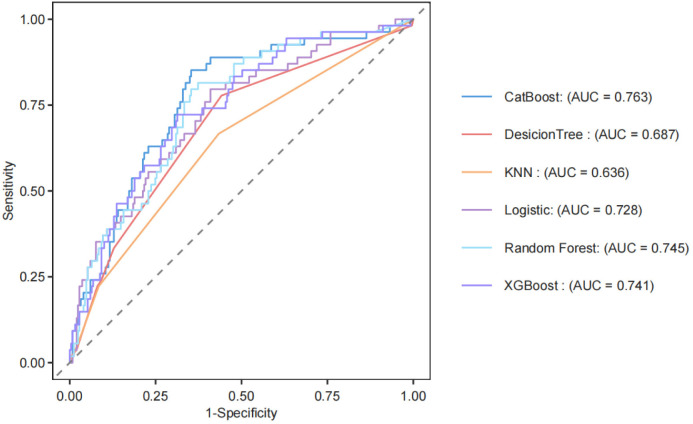
Receiver operator characteristic (ROC) curves of the machine learning algorithms. CatBoost: Categorical Boosting; DecisionTree: Decision Tree Learner Plus; KNN: K-Nearest Neighbor Learner Plus; Logistic: Logistic Classification Learner Plus; XGBoost: eXtreme Gradient Boosting; Random Forest: Random Forest Learner Plus; AUC: area under the curve.

Fig 5 presents the ROC curves for six models, with their performance indicated by the AUC values. The models are ranked in descending order of performance as follows: CatBoost (AUC = 0.763), Random Forest (AUC = 0.745), XGBoost (AUC = 0.741), Logistic (AUC = 0.728), Decision Tree (AUC = 0.687), and KNN (AUC = 0.636). The CatBoost model demonstrated the best performance. The calibration curves for the CatBoost, Random Forest, XGBoost, and Logistic models closely align with the reference line, indicating their excellent predictive performance.

### Boruta algorithm

**Fig 6:**
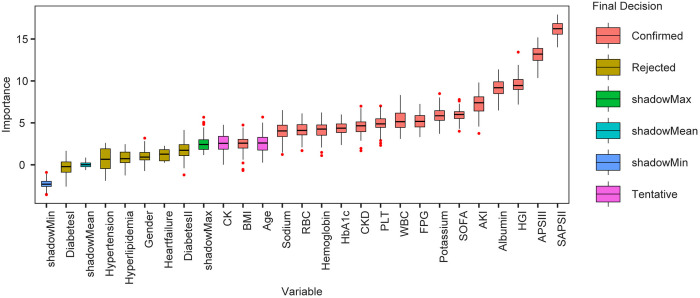
Selection of features using Boruta’s technique. Each variable’s name appears on the horizontal axis, while its Z-value appears on the vertical axis, which reflects the degree of importance of the variables. Box plots are used to depict the distribution of the Z values for variables. Variables in the red frame are crucial; those in the purple frame have provisional qualities; and variables in the yellow frame are inconsequential.

Fig 6 presents the Boruta algorithm based on CatBoost, which ranks the importance of variables for further selection. This process identified 16 variables deemed suitable, as indicated by the red region, which were utilized in the creation of the machine learning model.

### Shapley additive explanations

**Fig 7:**
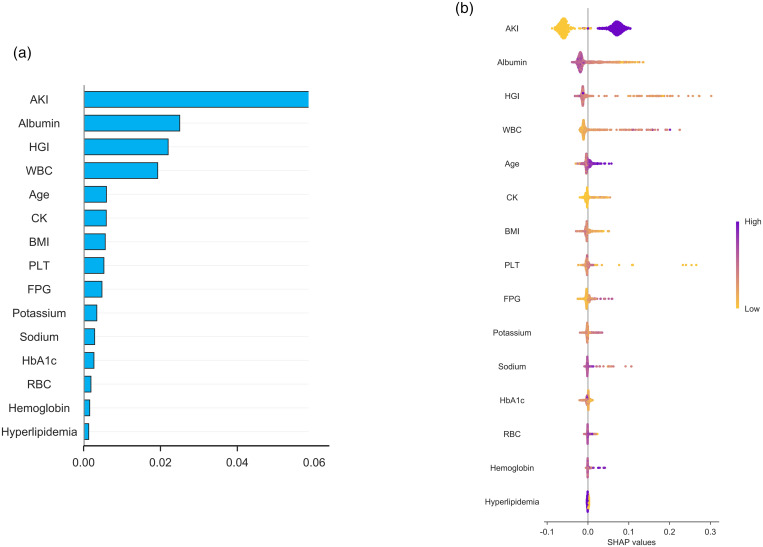
Shapley Additive Explanations (SHAP) summary plot. (A) The significance of SHAP features as evaluated by the mean absolute Shapley value. (B) SHAP Bees Plot, The SHAP values are distributed along the horizontal axis, where positive values indicate that the feature increases the predicted outcome, and negative values indicate that it decreases the predicted outcome. The color gradient ranges from yellow (representing lower values) to purple (representing higher values) of the feature.

The 15 clinical features are displayed in Fig 7A, ranked by their mean absolute SHAP values. The features are organized in descending order of importance, with the top feature having the greatest impact on the predictive model; longer bars indicate a larger influence. Among them, AKI has the highest importance, followed by albumin and HGI. Fig 7B presents the summary plot of variables in the CatBoost model. Features specific to mortality include AKI, WBC, age, sodium, and hemoglobin, each with positive SHAP values, indicating their predictive power for mortality. The SHAP values of HGI were broadly dispersed and primarily positive, indicating that an increase in HGI was connected with an increase in the projected results. Fig 7 shows the features associated with increased mortality risk, ranked in descending order of importance: AKI, albumin, HGI, WBC, age, CK, BMI, PLT, FPG, potassium, sodium, HbA1c, RBC, hemoglobin, and hypertension. Although AKI demonstrates high predictive value in the model, HGI still highlights its unique advantages, as evidenced by the multivariate Cox regression model (Table 1). As a comprehensive indicator, HGI not only reflects acute fluctuations in glucose metabolism but also incorporates long-term blood glucose control, providing a more comprehensive assessment of the patient’s metabolic state. The independent advantage of HGI is its ability to reveal risks associated with metabolic disorders such as insulin resistance and endothelial dysfunction, which may not be fully captured in AKI [19]. Despite the more significant direct association between AKI and mortality, HGI remains an important supplementary indicator in clinical judgment.

## Discussion

This study found that low HGI is strongly related to the short-term prognosis of individuals with AMI, whereas high HGI is not. To exclude the influence of diabetes on outcomes, we adjusted for covariates such as BMI, WBC, and diabetes, and the results remained consistent, demonstrating that the conclusion is valid for diabetic and non-diabetic people. As a result, HGI could be an independent indicator of poor prognosis in AMI patients. This finding not only enriches the evidence of HGI in the prognosis of cardiovascular diseases but also provides a new potential indicator for the prognosis of AMI patients.

For the past several years, the HGI has been recommended as a key indication for predicting the risk of microvascular problems [20–22]. While conventional predictors of AMI mortality (e.g., age, hemodynamic parameters, HbA1c) are well-established, these static clinical indicators may fail to accurately capture dynamic metabolic dysregulation during acute coronary events. This limitation is particularly striking given the clear association between hyperglycemia and excessive blood glucose control (<7.8 mmol/L) with AMI outcomes [23,24], as both extremes are associated with higher mortality and vascular complications. Lin et al. demonstrated that elevated HGI values exhibit an inverse correlation with the risk of CVD mortality. However, this relationship is only partially dependent on cardiorespiratory fitness levels [25]. Qing et al. found that HGI is related to CVD and risk of all-cause mortality in hypertensive individuals [26]. Wang et al. discovered that in people without CVD, HGI can be used to identify those who are at risk of subclinical myocardial injury (SC-MI), and HGI has been shown to be an independent predictor of SC-MI [27]. Furthermore, HGI exhibits a U-shaped relationship with the incidence of serious adverse cardiac events, with lower HGI indicating an elevated risk of cardiovascular mortality [28]. These studies show a correlation between HGI and unfavorable outcomes in cardiovascular patients. Our study not only confirms prior findings, but also shows that the HGI can give independent predictive value even after accounting for established AMI mortality predictors. Unlike traditional single biomarkers, the HGI, which is computed from both real and measured HbA1c values, takes into account both acute stress reactions and chronic metabolic dysfunction. Furthermore, unlike biomarkers, which require specialized testing, the HGI is easily derived from current clinical data, increasing its potential for routine use in risk assessment. While the HGI cannot replace proven prognostic criteria, it does provide a clinically relevant tool for identifying patients with metabolic vulnerabilities that may be missed by conventional approaches. The HGI should be used in conjunction with existing risk scores and biomarkers to create a more thorough risk assessment. Therefore, our study, based on data from the MIMIC-IV database, created a predictive model to analyze the impact of the HGI on mortality in patients with AMI using machine learning techniques.

Hemoglobin glycation index (HGI) has a complex molecular association with the pathogenesis of acute myocardial infarction (AMI), and the underlying mechanism may be closely related to insulin resistance (IR) [29]. HGI, as an important biomarker of cardiovascular disease, may affect several pathologic and physiologic processes such as endothelial function, vascular stiffness, lipid metabolism, oxidative stress, and inflammatory response by reflecting the state of IR [30]. The study findings demonstrate several important connections between HGI and clinical outcomes in AMI patients. First, we observed substantial correlations between low HGI and all-cause mortality, high AKI prevalence, and elevated white blood cell counts in all HGI subgroups. These comorbid conditions likely contribute to increased mortality risk by exacerbating systemic inflammation and endothelial dysfunction, potentially creating synergistic effects with dysglycemia. This correlation was more pronounced in nondiabetic patients, suggesting that HGI may capture features of metabolic abnormalities not captured by traditional glycemic indicators. Second, baseline data revealed substantial differences in SOFA, APSIII, and SAPSII scores across patients. In various HGI groups, demonstrating a close relationship between HGI and disease severity. Third, comparative analysis showed the Q1 and Q4 groups had considerably greater FPG levels compared to the Q2 and Q3 groups. This suggests that FPG is not only a strong predictor of mortality in AMI patients but also that elevated levels are related to poor prognosis in AMI [31,32]. Additionally, the WBC counts in the Q1 and Q4 groups had much higher values than the Q2 and Q3 groups, suggesting that both high and low HGI are linked with a higher inflammatory load. The current study suggests that HGI may act as a glucose metabolism phenotype reflecting the levels of inflammatory markers such as ultrasensitive C-reactive protein, erythrocyte sedimentation rate, complement C3, leukocyte count and fibrinogen [33]. The low HGI state may reflect an individual’s unique profile of disturbed glucose metabolism, a metabolic abnormality that can lead to an abnormal up-regulation of the activity of the NADPH oxidase system, resulting in a high level of reactive oxygen species (ROS) production, which then triggers an oxidative stress response [34]. This mechanism not only compromises vascular endothelial and cardiomyocyte function, but also stimulates the release of inflammatory factors through the activation of the NF-κB signaling pathway, hence aggravating atherosclerotic plaque development and instability [35]. Meanwhile, patients with low HGI may have more severe vascular endothelial dysfunction, as demonstrated by decreased nitric oxide synthesis, decreased endothelial cell function, and impaired vascular repair due to aberrant eNOS activity [34]. Together, these pathological alterations cause greater coronary plaque instability, microcirculatory dysfunction, and lower myocardial repair capacity, resulting in more severe myocardial injury and mortality. These processes interact with one another, forming the molecular basis for HGI to increase AMI development, presenting a possible target for early therapeutic intervention.

In the Kaplan-Meier survival analysis, lower HGI was strongly linked with mortality in AMI patients, but higher HGI had no significant relationship with short-term mortality. In the Cox proportional hazards model, the correlation between low HGI and outcome events was consistent with the core outcome. The P-values for The P-values for the HGI group trend were all < 0.05, indicating a significant linear trend between HGI and mortality. Nevertheless, the linear trend test might only estimate the overall trend between HGI and mortality but is unable to represent any potential non-linear relationships. To detect further about the relationship between mortality and HGI, we employed a RCS analysis. The result of the RCS analysis showed a significant U-shaped association between HGI and mortality. The U-shaped association means that the impact of HGI on mortality is not a simple linear association, but rather there is an “optimal range” in which the risk of death is lower. The RCS analysis, through the fitted curve, portrays more accurately the shape of the effect of HGI on mortality. In the subgroup analysis, low HGI levels were strongly related to outcome events, particularly among those with hypertension, AKI, non-CKD, non-diabetes, aged 65 and above, and BMI ≥ 25. This shows that advanced age, increased BMI, and hypertension may be negative risk factors for AMI. Interestingly, HGI among patients with AMI and diabetes appeared to be unrelated to all-cause mortality, which contradicted prior findings. In addition, we used SHAP and Brouta plots to perform feature analysis and assess the significance of HGI as a feature in the outcome prediction model. In conclusion, our study shows that HGI is strongly linked with mortality in patients with AMI.

However, this study has certain limitations. First, due to limitations in the data included, complete clinical diagnostic information may not have been extracted, and the consideration of confounding factors affecting mortality may be insufficient. Second, HGI is not dynamically monitored, and the emergence and development of an AMI may interfere with lipid metabolism and induce changes in blood glucose levels. Therefore, when assessing the prognosis of individual patients, it is necessary to consider the specific level of HGI, rather than just its categorization. Lastly, the HGI in this study was estimated using the MIMIC-IV database, which may not be generalizable to different populations. Therefore, it is recommended to retrieve data from various large databases to establish regression models for calculating HGI in different population types. Despite these limitations, our study provides valuable insights into the relationship between HGI and AMI prognosis, highlighting the potential of HGI as a prognostic indicator in clinical practice. Future research should focus on validating these findings in diverse populations and exploring the underlying mechanisms linking HGI to AMI outcomes.

Our results in the HGI and AMI studies differ from previous research that found a strong correlation between high HGI and CVD, whereas our results suggest a significant association between low HGI and short-term mortality in AMI patients. The likelihood of a bad prognosis rises with a lower HGI. As a result, HGI can be utilized to predict the short-term prognosis of AMI patients. Lastly, HGI’s ability to forecast short-term mortality in AMI may vary across different populations, necessitating validation through multicenter, prospective studies.

## Supporting information

S1 Table Comparison of the patients’ baseline data based on the HGI. (DOCX)
