# Supplementary material for: Association between hemoglobin glycation index and 28-day all-cause mortality in acute myocardial infarction patients: Analysis of the MIMIC-IV database
Source: PLoS One. 2025 Sep 2;20(9):e0330819. doi: 10.1371/journal.pone.0330819 (PMC12404404; doi:10.1371/journal.pone.0330819)
Supplement: S1 Table — (DOCX) [file pone.0330819.s001.docx]

**S1 Table** Comparison of the patients' baseline data based on the HGI

| **Variable** | **Total** | **Q1 (<-0.81)** | **Q2 (-0.81,-0.35)** | **Q3 (-0.35,0.32)** | **Q4 (>0.32)** | **P-value** |
| --- | --- | --- | --- | --- | --- | --- |
| N | 1,008 | 252 | 252 | 252 | 252 |  |
| Age (years) | 67.20 (12.78) | 67.02 (12.98) | 65.92 (13.50) | 68.77 (12.41) | 67.09 (12.11) | 0.12 |
| BMI (kg/m²) | 29.09 (6.47) | 28.19 (6.63) | 28.50 (5.29) | 29.94 (6.96) | 29.74 (6.72) | **<0.001** |
| Gender (%) |  |  |  |  |  | 0.24 |
| Female | 321.00 (31.85%) | 89.00 (35.32%) | 68.00 (26.98%) | 82.00 (32.54%) | 82.00 (32.54%) |  |
| Male | 687.00 (68.15%) | 163.00 (64.68%) | 184.00 (73.02%) | 170.00 (67.46%) | 170.00 (67.46%) |  |
| SOFA score | 4.71 (3.44) | 5.94 (3.88) | 4.45 (3.19) | 4.02 (3.18) | 4.43 (3.17) | **<0.001** |
| APSIII score | 44.21 (20.01) | 50.55 (22.83) | 39.75 (17.06) | 40.03 (17.42) | 46.50 (20.17) | **<0.001** |
| SAPSII score | 36.29 (13.19) | 40.46 (14.12) | 34.00 (12.16) | 34.59 (12.80) | 36.14 (12.71) | **<0.001** |
| HGB (g/dL) | 11.82 (2.48) | 11.57 (2.75) | 12.24 (2.51) | 12.00 (2.27) | 11.47 (2.30) | **<0.001** |
| PLT ( 10^9/L) | 223.58 (84.45) | 226.99 (87.25) | 223.05 (77.61) | 228.52 (90.69) | 215.76 (81.57) | 0.38 |
| RBC (10^9/L) | 3.95 (0.80) | 3.78 (0.86) | 4.05 (0.81) | 4.04 (0.75) | 3.96 (0.77) | **<0.001** |
| WBC (10^9/L) | 13.03 (6.53) | 14.63 (7.63) | 12.88 (6.84) | 11.63 (5.02) | 12.96 (6.03) | **<0.001** |
| HbA1c (%) | 6.55 (1.75) | 5.39 (0.58) | 5.68 (0.44) | 6.22 (0.60) | 8.90 (1.90) | **<0.001** |
| FPG (mg/dL) | 182.11 (107.53) | 211.60 (139.85) | 142.96 (56.38) | 147.50 (72.00) | 226.38 (113.51) | **<0.001** |
| HGI | 0.00 (1.55) | -1.38 (0.86) | -0.58 (0.13) | -0.06 (0.19) | 2.03 (1.59) | **<0.001** |
| Albumin (g/dL) | 3.37 (0.57) | 3.28 (0.59) | 3.43 (0.58) | 3.47 (0.54) | 3.30 (0.55) | **<0.001** |
| Potassium (mmol/L) | 4.29 (0.72) | 4.28 (0.77) | 4.26 (0.67) | 4.23 (0.70) | 4.37 (0.74) | 0.20 |
| Sodium (mmol/L) | 137.73 (4.88) | 137.19 (5.62) | 137.59 (4.69) | 138.21 (4.03) | 137.92 (5.03) | **0.09** |
| CK (IU/L) | 1,447.39 (2,861.70) | 2,041.69 (4,213.84) | 1,531.17 (2,432.32) | 1,043.39 (1,400.35) | 1,173.32 (2,573.06) | **0.02** |
| Hypertension (%) |  |  |  |  |  | **0.02** |
| NO | 644.00 (63.89%) | 176.00 (69.84%) | 154.00 (61.11%) | 145.00 (57.54%) | 169.00 (67.06%) |  |
| YES | 364.00 (36.11%) | 76.00 (30.16%) | 98.00 (38.89%) | 107.00 (42.46%) | 83.00 (32.94%) |  |
| AKI (%) |  |  |  |  |  | **<0.001** |
| NO | 542.00 (53.77%) | 126.00 (50.00%) | 151.00 (59.92%) | 159.00 (63.10%) | 106.00 (42.06%) |  |
| YES | 466.00 (46.23%) | 126.00 (50.00%) | 101.00 (40.08%) | 93.00 (36.90%) | 146.00 (57.94%) |  |
| CKD(%) |  |  |  |  |  | **<0.001** |
| NO | 766.00 (75.99%) | 196.00 (77.78%) | 211.00 (83.73%) | 192.00 (76.19%) | 167.00 (66.27%) |  |
| YES | 242.00 (24.01%) | 56.00 (22.22%) | 41.00 (16.27%) | 60.00 (23.81%) | 85.00 (33.73%) |  |
| Diabetes II (%) |  |  |  |  |  | **<0.001** |
| NO | 616.00 (61.11%) | 200.00 (79.37%) | 210.00 (83.33%) | 166.00 (65.87%) | 40.00 (15.87%) |  |
| YES | 392.00 (38.89%) | 52.00 (20.63%) | 42.00 (16.67%) | 86.00 (34.13%) | 212.00 (84.13%) |  |
| Diabetes I (%) |  |  |  |  |  | **<0.001** |
| NO | 981.00 (97.32%) | 248.00 (98.41%) | 249.00 (98.81%) | 250.00 (99.21%) | 234.00 (92.86%) |  |
| YES | 27.00 (2.68%) | 4.00 (1.59%) | 3.00 (1.19%) | 2.00 (0.79%) | 18.00 (7.14%) |  |
| Hyperlipidemia (%) |  |  |  |  |  | **<0.001** |
| NO | 481.00 (47.72%) | 133.00 (52.78%) | 136.00 (53.97%) | 115.00 (45.63%) | 97.00 (38.49%) |  |
| YES | 527.00 (52.28%) | 119.00 (47.22%) | 116.00 (46.03%) | 137.00 (54.37%) | 155.00 (61.51%) |  |
| Heart failure (%) |  |  |  |  |  | **0.02** |
| NO | 476.00 (47.22%) | 106.00 (42.06%) | 124.00 (49.21%) | 137.00 (54.37%) | 109.00 (43.25%) |  |
| YES | 532.00 (52.78%) | 146.00 (57.94%) | 128.00 (50.79%) | 115.00 (45.63%) | 143.00 (56.75%) |  |
| CHD (%) |  |  |  |  |  |  |
| YES | 1,008.00 (100.00%) | 252.00 (100.00%) | 252.00 (100.00%) | 252.00 (100.00%) | 252.00 (100.00%) |  |
| Continuous numerical variables are expressed as medians (interquartile spacing) and categorical variables are expressed as numbers (percentages) BMI:Body Mass Index,SOFA:Sepsis-Organ Failure Assessment Score,APSIII:Acute Physiology Score III, SAPS II:Simplified Acute Physiology Score II,WBC:white blood cells, RBC:red blood cells, PLT:platelets, HGB:hemoglobin, FPG:fasting plasma glucose,HbA1c:glycated hemoglobin,HGI:hemoglobin glycation index,CK:Creatine Kinase,AKI:acute kidney injury,CKD:Chronic kidney disease,Diabetes II:Diabetes mellitus type 2,Diabetes I:diabetes mellitus type 1,MI:Myocardial infarction,CHD:Coronary Heart Disease. | | | | | | |
